# OVH‐guided planning for superior heart and lung sparing in breast cancer radiotherapy

**DOI:** 10.1002/acm2.70513

**Published:** 2026-03-08

**Authors:** Hao Lei, Dan Li, Wei Wei, Hongmei Zheng, Xinhong Wu, Xudong Xue

**Affiliations:** ^1^ Department of Radiation Oncology Hubei Cancer Hospital Tongji Medical College Huazhong University of Science and Technology Wuhan Hubei China; ^2^ Breast cancer center Hubei Cancer Hospital Tongji Medical College Huazhong University of Science and Technology National key clinical specialty construction discipline Hubei Provincial Clinical Research Center for Breast Cancer Wuhan Clinical Research Center for Breast Cancer Wuhan Hubei China

**Keywords:** automated planning, breast cancer, DVH, OVH, radiotherapy

## Abstract

**Background and purpose:**

Manual planning in breast cancer radiotherapy is often time‐consuming and operator‐dependent, leading to inconsistencies in plan quality. This study validated an automated workflow using overlap volume histograms (OVH) to predict patient‐specific dose–volume histogram (DVH) constraints, aiming to enhance cardiopulmonary sparing and planning efficiency.

**Materials and methods:**

A historical database of 322 patients was stratified into four groups: left/right post‐mastectomy radiotherapy (PMRMRT) and left/right breast‐conserving radiotherapy (BCRT). Linear regression models were established to correlate OVH‐derived geometric metrics (*L_x_*) with corresponding DVH‐based dose constraints (*D_x_*). These predictive models were integrated into the Monaco treatment planning system via a custom Python script to provide an improved automated planning workflow. The workflow's performance was prospectively validated on 80 independent testing cases (20 per group). Automated plans were generated using the predicted constraints and compared dosimetrically against clinically approved manual plans.

**Results:**

Significant linear correlations were observed between *L_x_* and *D_x_* for all OARs (*r^2^* = 0.51–0.72, *p* < 0.001). In the PMRMRT testing cohorts, the automated workflow significantly reduced doses to the heart and ipsilateral lung compared to manual planning (*p* < 0.05). For left‐sided PMRMRT, the heart dose was reduced by 15.6% (*D*_10_), 18.7% (*D*_5_), and 9.8% (*D*_mean_), while the ipsilateral lung dose decreased by up to 6.3% (*D*_mean_). In BCRT cases, automated plans were not significant improved compared to manual plans. Importantly, all automated plans maintained target volume coverage and dose homogeneity comparable to manual plans (*p* > 0.05).

**Conclusion:**

The OVH‐based framework effectively translated anatomy into achievable objectives, significantly improving heart and lung sparing for complex PMRMRT cases while streamlining clinical workflows.

## 1 INTRODUCTION

Breast cancer stands as one of the most common malignant tumors among women globally, with its incidence and mortality rates continuing to rise. According to a report published in the Lancet in 2024, the global number of breast cancer cases increase from 2.3 million in 2020 to over 3 million by 2040.^1^ In China, breast cancer ranks first among female malignancies in terms of incidence rate and fifth in mortality rates.^2^ Radiotherapy, as an essential component of comprehensive breast cancer treatment, is primarily employed to reduce the risk of local recurrence and to prolong survival in patients following mastectomy. It also serves as a crucial palliative treatment modality for individuals with locally advanced or metastatic disease.

Intensity‐modulated radiotherapy (IMRT), volumetric‐modulated arc therapy (VMAT), and deep‐inspiration breath‐hold (DIBH) are now the prevailing techniques for breast‐cancer radiotherapy. These techniques, allow improved dose conformity, better PTV homogeneity, and improved sparing of OARs. Moreover, the inverse planning process enables optimization of specific dose–volume objectives, unlike the forward planning typical for three‐dimensional conformal radiotherapy (3D‐CRT) plans. VMAT generally outperforms other technique in improving dose conformity. However, the increased low‐dose bath to normal tissue remains a relevant concern for rotational techniques, as VMAT often results in higher mean dose to contralateral OARs compared with conformal methods.

Achieving an optimal IMRT plan involves a labor‐intensive, iterative trial‐and‐error process.^3^ The final plan quality is highly dependent on the planner's experience and manual adjustments, requiring repeated modifications of objective functions and their associated weights until clinically acceptable dose–volume‐histogram (DVH) metrics and three‐dimensional dose distributions are attained. In breast radiotherapy planning, we should balance the adequate target coverage with sparing of organs at risk (OARs), such as the heart and ipsilateral lung. Moreover, in cases requiring regional nodal irradiation or involving complex anatomical geometries, the conventional manual approach becomes increasingly inefficient and less reproducible.^4^

To address these limitations, geometry‐based metrics have been proposed to guide and automate radiotherapy planning. Among them, the overlap volume histogram (OVH) has emerged as a promising quantitative descriptor that captures the spatial relationship between the target volume and surrounding organs.^5^, ^6^ The OVH represents the fractional volume of an OAR that lies within a given distance from the planning target volume (PTV), providing a patient‐specific geometric context for dose estimation and optimization. As mentioned before, one of the major challenges in radiotherapy lies in the mathematical definition of DVH objectives that can effectively balance the trade‐off between PTV coverage and OAR sparing. The OVH‐based approach has the ability to predict the likely OAR DVHs from a geometrical perspective. Previous studies have shown that OVH predicted achievable dose–volume constraints and served as a foundation for knowledge‐based or atlas‐based planning frameworks.^7^, ^8^, ^9^, ^10^

This study aims to develop and validate an automated planning method based on the OVH framework. By quantifying the geometric proximity between the PTV and heart or lungs, the proposed approach seeks to generate predictive dose–volume relationships that can guide individualized plan optimization. Specifically, this work focuses on leveraging historical planning data to build OVH‐dose models, which can then be used to automatically predict achievable dose constraints and generate new treatment plans with minimal manual intervention.

## 2 METHODS AND MATERIALS

### 2.1 Patients and radiotherapy planning

In this retrospective study, a total of 322 breast cancer patients were enrolled. The cohort comprised patients who underwent either breast‐conserving radiotherapy (BCRT) or post‐modified radical mastectomy radiotherapy (PMRMRT). Among them, 164 had left‐sided disease (93 treated with PMRMRT and 71 with BCRT) and 158 had right‐sided disease (104 treated with PMRMRT and 54 with BCRT). For testing model, a separate test set consisting of 80 cases (20 left‐sided PMRMRT plans, 20 left‐sided BCRT plans, 20 right‐sided PMRMRT plans and 20 right‐sided BCRT plans) was used to evaluate the accuracy of the prediction model. Patients who received PMRMRT were treated with a conventional fractionation regimen of 50 Gy in 25 fractions. In contrast, BCT patients predominantly received a hypofractionated regimen of (2.7–2.9) Gy × 15 fractions, followed by a sequential boost of (2.0–2.9) Gy × (3–5) fractions. The clinical target volume (CTV) was isotropically expanded 5 mm to generate the PTV. In our center, the internal mammary lymph node area is generally not delineated separately but is considered as part of CTV. The prescription was also 50 Gy in 25 fractions.

In terms of treatment planning, we utilized IMRT or VMAT techniques for the design of these radiotherapy plans for breast cancer. For BCRT, due to the involvement of the chest wall alone, we employed multiple tangential fields or partial arcs. For PMRMRT patients, the target volumes were typically divided into two subregions: the supraclavicular region and the chest wall. The chest wall was irradiated using tangential fields, while the supraclavicular region was treated using either fixed beams (typically at 180°, 330°, and 30°) or partial arcs (e.g., 180°–210° and 330°–0° for right‐sided cases; 0°–30° and 150°–180° for left‐sided cases). The details of the VMAT or IMRT techniques used in this study were shown in S1 figure of supplementary materials.

All treatment plans were calculated using a grid spacing of 0.3 cm, employing the Monte Carlo Photon dose calculation algorithm (Monaco TPS Version 6.0.11) with a statistical uncertainty of 1% per calculation. All the IMRT or VMAT plans were delivered on an Elekta linear accelerator (Elekta AB, Stockholm, Sweden) equipped with agility 160 leaves MLC collimator.^11^ 6 MV or 6 MV flattening filter‐free (FFF) photon beams were used in this study. All patients were positioned supine using a thermoplastic mask or vacuum cushion for immobilization during CT simulation and treatment.

### 2.2 OVH definition

The OVH descriptor is defined as follows^5^, ^6^:

(1) $$OVH_{O,T}\left(d\right)=\frac{\left|\left\{p\in O|d(p,T)\leq d\right\}\right|}{\left|O\right|}$$

where T is tumor target volume, O represent an OAR of interest; |O| is the volume of the OAR; *d*(*p*, *T*) represents the minimum Euclidean distance from voxel *p* to the boundary of the target volume *T*; |{p∈O|d(p,T)≤d}| represents a subset of OAR whose distance to the tumor is less than *d*.

To quantitatively characterize the geometric relationship between targets and OARs, we computed the OVH curves separately for the ipsilateral lung and heart in breast cancer cases using an in‐house developed Python script.

### 2.3 Relationship between OVH and DVH metrics

In this study, the correlations between OVH metrics (*L_x_* and *D_x_*) were established using linear regression analysis across corresponding points. The x here denotes the volume. For instance, considering the clinical constraint for the ipsilateral lung (*V*_5Gy_≤50%), the metric *L*_50_ was defined as the isotropic expansion distance of the PTV required to encompass 50% of the lung volume. Correspondingly, *D*_50_ represents the dose received by 50% of the lung volume (*D*_50%_), serving as an indicator of lung sparing. This methodology is schematically illustrated in Figure S2 (Supplementary Materials). Similarly, *L*_25_ and *L*_35_ were determined for the ipsilateral lung, corresponding to 25% and 35% volume overlaps, respectively, with their associated *D*_25_ and *D*_35_​ values extracted from the DVH. For left‐sided breast cancer cases, heart‐specific metrics (*L*_10_–*D*_10_, and L_5_–D_5_) were also calculated. All specific dose constraints for the ipsilateral lung and heart utilized in this study are summarized in Table 1.

**TABLE 1:** Dose constraints of breast cancer radiotherapy in this study.

Organs at risk	Conventional fractionation / hypofractionation
Left lung for left breast cancer	*V*_5Gy_ ≤ 50%
	*V*_10Gy_ ≤ 35%
	*V*_20Gy_ ≤ 25%
Heart for left breast cancer	*V*_20Gy_ ≤ 10%
	*V*_40Gy_ ≤ 5%
	*D*_mean_≤6∼8 Gy
Right lung for right breast cancer	*V*_5Gy_ ≤ 50%
	*V*_10Gy_ ≤ 35%
	*V*_20Gy_ ≤ 25%

### 2.4 Workflow of automated treatment planning

**FIGURE 1:**
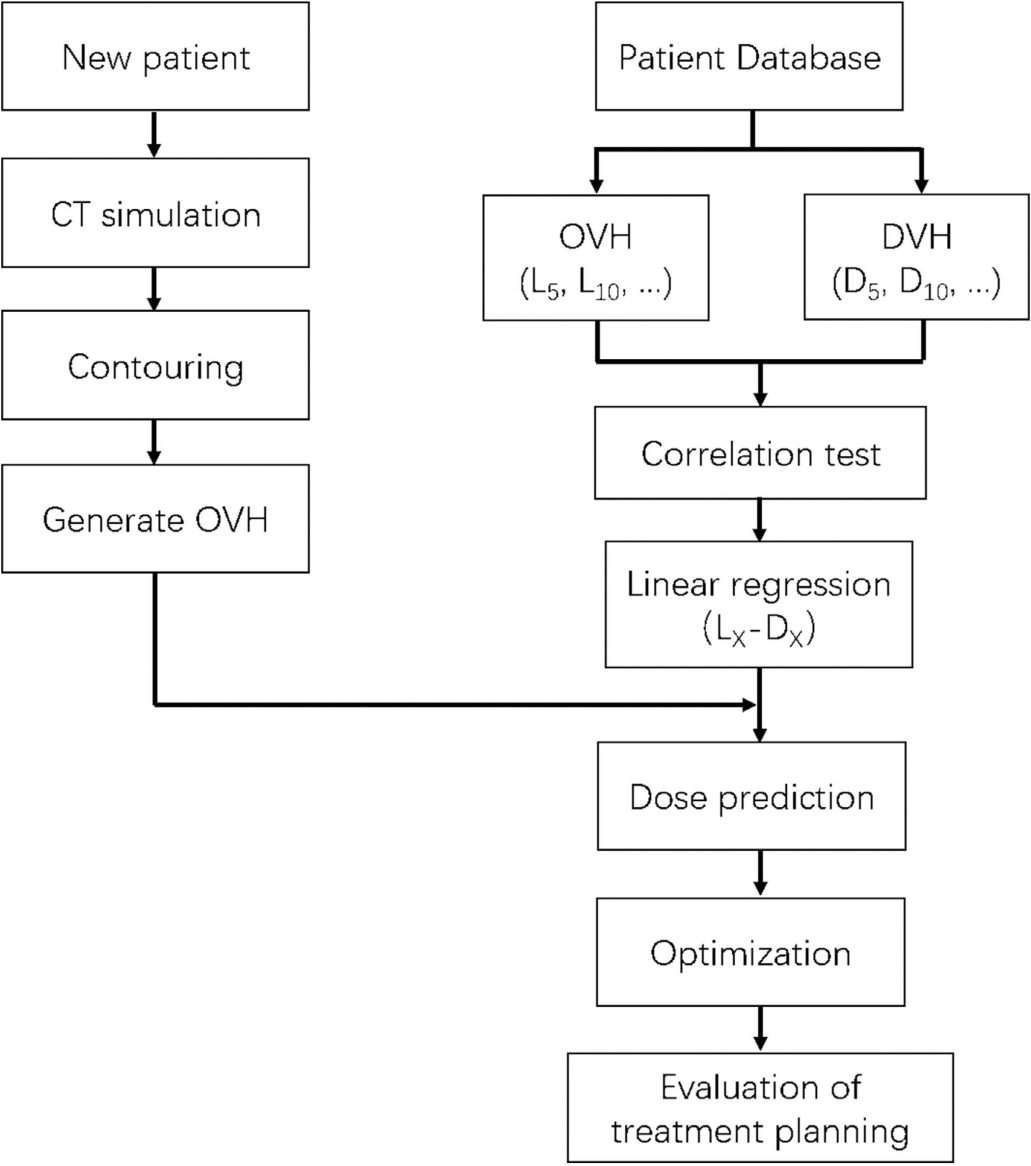
Schematic workflow of the automated treatment planning process integrating OVH and DVH predictive modeling.

In this study, we developed an automated workflow utilizing OVH and DVH to streamline treatment planning for breast cancer radiotherapy (Figure 1). First, a historical patient database (164 left‐sided breast patients and 158 right‐sided breast patients) was established to derive linear regression models correlating OVH‐based metrics (*L_x_*) with their corresponding DVH‐based dose constraints (*D_x_*), where x represents a specific fractional volume of an organ at risk (OAR) (e.g., 50%). Second, once the OARs and PTV for a new patient were delineated, the OVH metrics were analyzed to determine the *L_x_* values. The predicted *D_x_* constraints were then calculated using the preestablished regression equations. Third, a custom Python script was employed to automatically integrate these *D_x_* values into the Monaco treatment planning template,^12^ where the “Overdose DVH” cost function was dynamically updated (e.g., *V*_4.5_ _Gy _< 50%). Finally, the dosimetrist applied the updated template for plan optimization, a process that continued iteratively until the predefined cost function objectives were satisfied.

The dosimetric results were compared with that from the manual optimization based on medical physicist or dosimetrist (40 left and 40 right breast cancer patients each). In addition to comparing doses to organs at risk (OARs), we also evaluated dosimetric parameters of PTV, including *D*_2_, *D*_98_, *D*_95_, as well as the conformity index (CI) and homogeneity index (HI). These metrics were used to comprehensively assess target dose coverage, uniformity, and conformity. The CI is calculated as:

(2) $$\mathrm{CI}={\left(TV_{P}\right)}^{2}/\left(V_{\mathrm{PTV}}\times V_{P}\right),$$

where TV_P_ denotes the volume of the target covered by the prescription dose, V_PTV_ is the volume of the PTV, and V_P_ is the total volume enclosed by the prescription isodose line. A CI value closer to 1 indicates better target conformity.

The HI is calculated as:

(3) $$\mathrm{HI}=\left(D_{2}-D_{98}\right)/D_{P},$$

where *D_P_* is the prescription dose. A HI value closer to 0 indicates better dose uniformity within the target volume.

### 2.5 Statistical analysis

We first employed the Pearson correlation test to assess the linear trend and its strength between *L_x_* and *D_x_*, followed by linear regression analysis to establish the quantitative relationship between *L_x_* and *D_x_*. The *p* value was obtained under the two‐tail paired condition at a 95% confidence level. The Pearson correlation test was performed using SPSS statistical software (Version 23, IBM Corporation), and the linear regression analysis using SciPy package.

## 3 RESULTS

**FIGURE 2:**
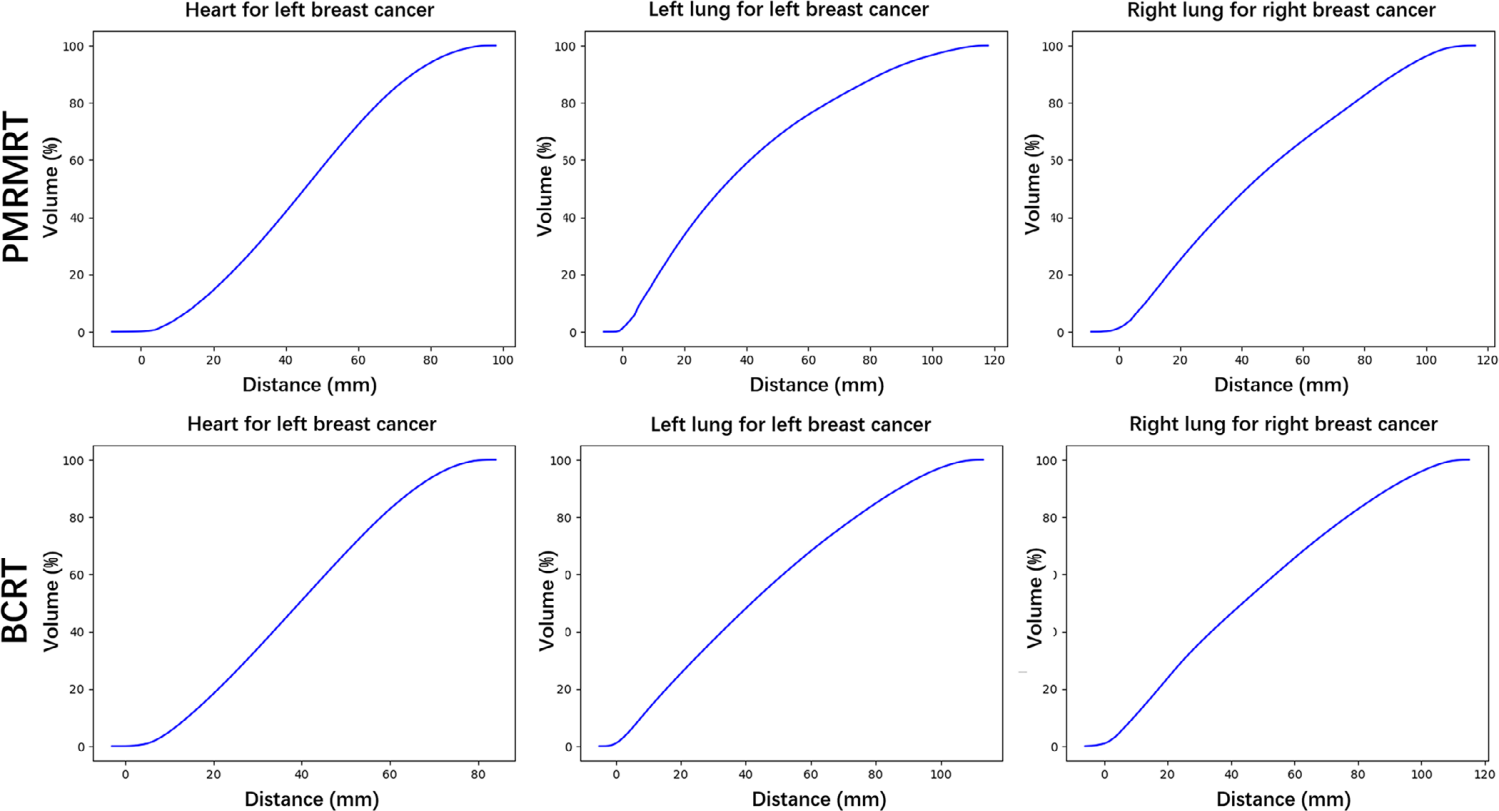
Representative OVH curves for the heart and ipsilateral lung in PMRMRT and BCRT cohorts, characterizing the geometric relationship between the PTV and OARs.

OVH curves were generated to quantitatively characterize the spatial relationship between the PTV and OARs. Figure 2 illustrates the representative OVH curves for the ipsilateral lung and heart from two left‐sided PMRMRT and BCRT patients, and two right‐sided PMRMRT and BCRT patients. For both the heart and the ipsilateral lung, a minimum isotropic expansion of 6 cm from the PTV was required to achieve complete volumetric overlap. Notably, due to its larger anatomical volume and broader spatial distribution compared to the heart, the ipsilateral lung required a significantly greater expansion distance for total coverage by the expanded PTV.

**FIGURE 3:**
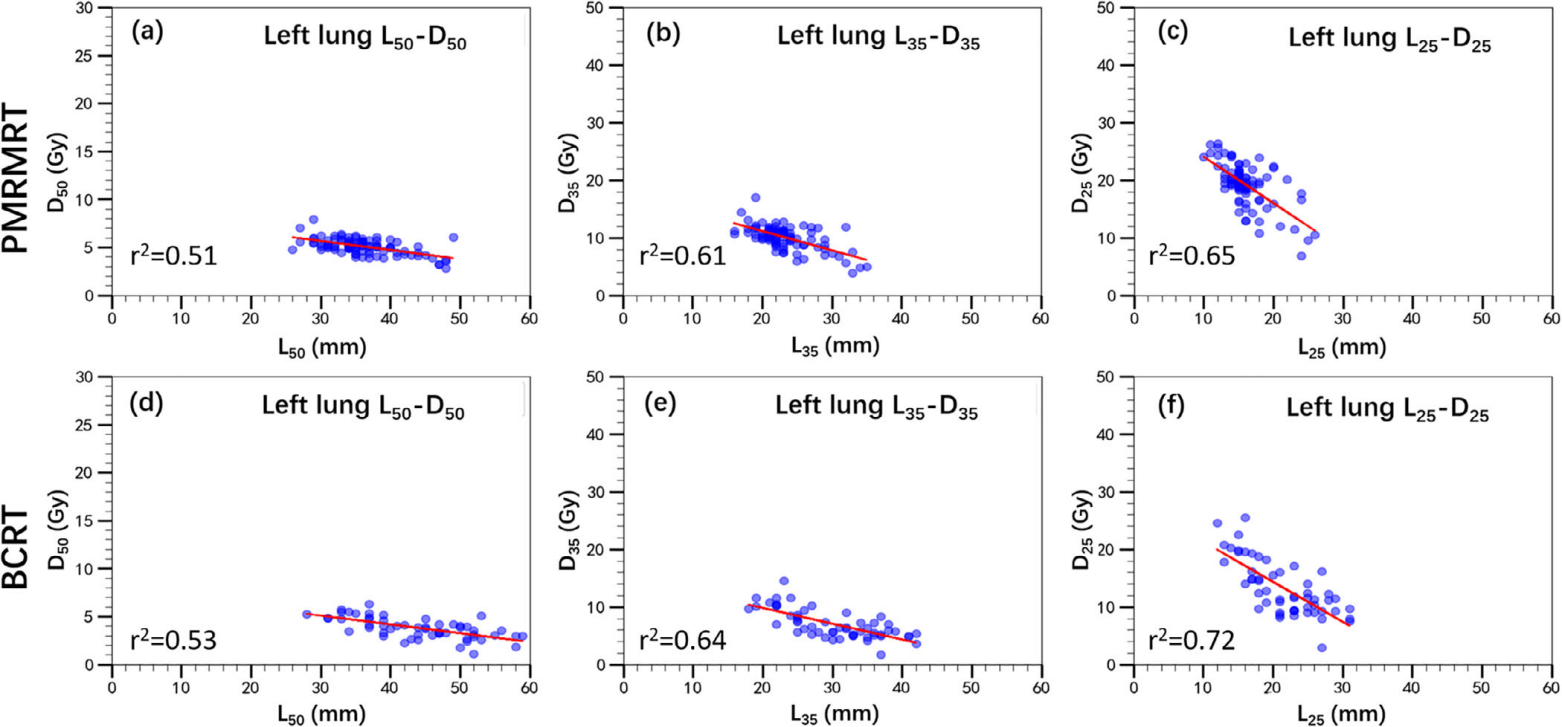
Correlations between OVH‐derived geometric metrics (*L_x_*) and DVH‐based dose constraints (*D_x_*) for the ipsilateral lung in left‐sided PMRMRT and BCRT patients. Red lines denote the linear regression fits.

**FIGURE 4:**
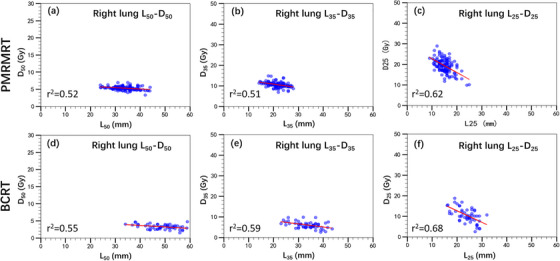
Correlations between *Lx* and *Dx* metrics for the ipsilateral lung in right‐sided PMRMRT and BCRT patients. Red lines denote the linear regression fits.

**FIGURE 5:**
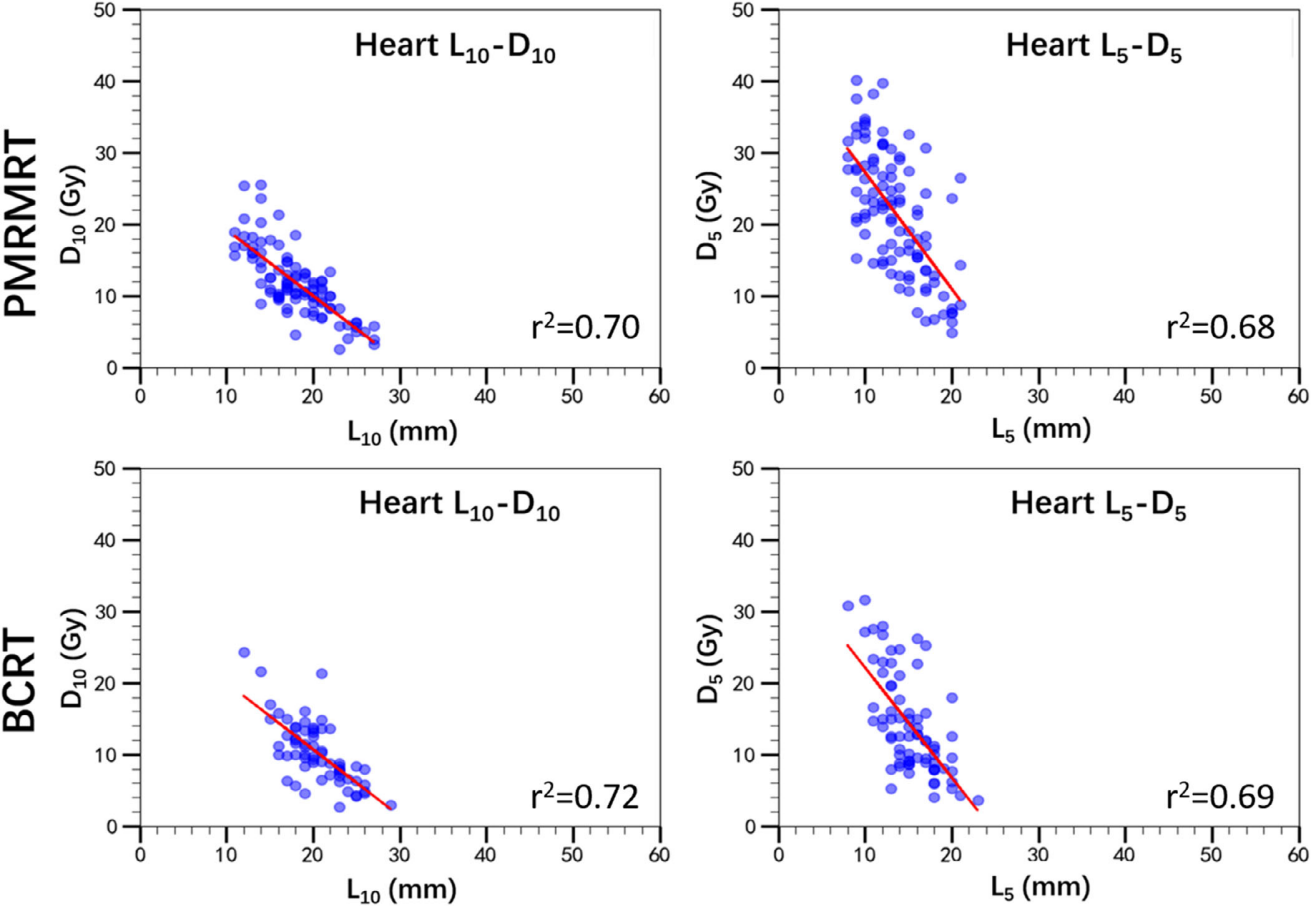
Correlations between *Lx* and *Dx* metrics for the heart in left‐sided PMRMRT and BCRT patients. Red lines denote the linear regression fits.

The relationships between *L_x_* and *D_x_* metrics were established through point‐wise linear regression analysis between the corresponding OVH and DVH curves. Scatter plots and the corresponding linear regression fits (indicated by red lines) for the ipsilateral lung (*D*_50_–*L*_50_, *D*_35_–*L*_35_, *D*_25_–*L*_25_​) and across both left‐ and right‐sided PMRMRT and BCRT cohorts are presented in Figures 3 and 4. Similarly, Figure 5 illustrates the heart‐specific correlations (*D*_10_–*L*_10_ and *D*_5_–*L*_5_) for left‐sided PMRMRT and BCRT cases.

Pearson correlation and linear regression analyses demonstrated significant positive correlations between *L_x_* and *D_x_* for both the ipsilateral lung and the heart. For left‐sided cases, the *r^2^* values for lung *D*_50_–*L*_50_, *D*_35_–*L*_35_ and *D*_25_–*L*_25_ were 0.51 versus 0.53, 0.61 versus 0.64, and 0.65 versus 0.72 for PMRMRT and BCRT, respectively. A consistent trend was observed in right‐sided cases, with *R^2^* values of 0.52 versus 0.55 (*D*_50_–*L*_50_), 0.51 vs. 0.59 (*D*_35_–*L*_35_), and 0.62 versus 0.68 (*D*_25_–*L*_25_). Notably, the heart in left‐sided breast cancer cases exhibited even stronger correlations, with *R^2^* values of 0.70 versus 0.72 for *D*_10_–*L*_10_ and 0.68 versus 0.69 for *D*_5_–*L*_5_, regardless of the treatment technique (PMRMRT or BCRT). All correlations reached statistical significance (*p* < 0.001, two‐tailed). These results indicate that *L_x_* metrics serve as robust predictors for ipsilateral lung and heart doses. The corresponding linear regression equations established for *D_x_*–*L_x_* prediction are summarized in Tables 2 and 3, which are subsequently utilized to facilitate automated radiotherapy planning.

**TABLE 2:** Linear regression of *D_x_* (Gy) and *L_x_* (mm) for PMRMRT.

	Variables	Equation	*r^2^*	*p*
Left breast cancer	Left lung			
	*D*_50_, *L*_50_	*D*_50 _= −0.05*L*_50_ + 6.88	0.51	<0.001
	*D*_35_, *L*_35_	*D*_35 _= −0.33*L*_35_ + 17.84	0.61	<0.001
	*D*_25_, *L*_25_	*D*_25 _= −0.80*L*_25_ + 32.08	0.65	<0.001
	Heart			
	*D*_10_, *L*_10_	*D*_10 _= −0.92*L*_10_ + 28.45	0.70	<0.001
	*D*_5_, *L*_5_	*D*_5 _= −1.63*L*_5_ + 43.62	0.68	<0.001
Right breast cancer	Right lung			
	*D*_50_, *L*_50_	*D*_50 _= −0.04*L*_50_ + 6.58	0.52	<0.001
	*D*_35_, *L*_35_	*D*_35 _= −0.17*L*_35_ + 14.17	0.51	<0.001
	*D*_25_, *L*_25_	*D*_25 _= −0.67L_25_ + 29.35	0.62	<0.001

**TABLE 3:** Linear regression of *D_x_* (Gy) and *L_x_* (mm) for BCRT.

	Variables	Equation	*r^2^*	p
Left breast cancer	Left lung			
	*D*_50_, *L*_50_	*D*_50 _= −0.04*L*_50_ + 4.99	0.53	<0.001
	*D*_35_, *L*_35_	*D*_35 _= −0.27*L*_35_ + 15.31	0.64	<0.001
	*D*_25_, *L*_25_	*D*_25 _= −0.70*L*_25_ + 28.28	0.72	<0.001
	Heart			
	*D*_10_, *L*_10_	*D*_10 _= −0.93*L*_10_ + 29.36	0.72	<0.001
	*D*_5_, *L*_5_	*D*_5 _= −1.54*L*_5_ + 37.57	0.69	<0.001
Right breast cancer	Right lung			
	*D*_50_, *L*_50_	*D*_50 _= −0.04*L*_50_ + 5.35	0.55	<0.001
	*D*_35_, *L*_35_	*D*_35 _= −0.15*L*_35_ + 11.04	0.59	<0.001
	*D*_25_, L_25_	*D*_25 _= −0.56*L*_25_ + 23.87	0.68	<0.001

The linear regression models were applied to an independent testing dataset comprising 80 cases across four cohorts to evaluate the feasibility of OVH‐guided automated treatment planning. Detailed dosimetric comparisons between manual plans and automated re‐plans are summarized in Tables 4, 5, 6, 7. For the left‐sided PMRMRT cohort, automated re‐planning significantly reduced the ipsilateral lung dose by 4.7% for *D*_50_ (*p* = 0.012), 1.6% for *D*_35_ (*p* = 0.055), 3.8% for *D*_25_ (*p* = 0.032), and 6.3% for *D*_mean_ (*p* = 0.013). Heart sparing was also substantially improved, with dose reductions of 15.6% for *D*_10_ (*p* = 0.003), 18.7% for *D*_5_ (*p* < 0.001), and 9.8% for *D*_mean_ (*p* < 0.001). Importantly, these reductions in OAR doses did not compromise PTV coverage or dose hot spots. For instance, *D*_95_, *D*_98_, and *D*_2_ remained comparable between manual and re‐plans (*D*_95_: 50.62 ± 0.81 Gy vs. 50.45 ± 0.76 Gy; *D*_98_: 49.96 ± 0.52 Gy vs. 49.79 ± 0.44 Gy; *D*_2_: 54.31 ± 1.33 Gy vs. 54.11 ± 1.33 Gy; all *p* > 0.05). The CI and HI also showed no significant changes. Similarly, in the right‐sided PMRMRT cohort, automated planning significantly decreased the ipsilateral lung dose (*p* < 0.05) while maintaining PTV consistency. For BCRT patients, since the OAR doses in the original clinical plans were already well below the constraints specified in Table 1, the automated re‐optimization showed no statistically significant differences compared to manual planning (*p* > 0.05, Tables 6 and 7).

**TABLE 4:** Dose–volume metrics for left lung, heart, and PTV in the manual, predicted, and re‐planning plans of left PMRMRT testing dataset.

Left PMRMRT	Dose metric	Manual	Predicted	Re‐planning
Left lung	*D*_50_ (Gy)	5.08 ± 0.66	4.93 ± 0.67 (*p *= 0.013)	4.84 ± 0.65 (*p *= 0.012)
	*D*_35_ (Gy)	10.40 ± 1.82	10.31 ± 1.41 (*p *= 0.061)	10.27 ± 1.51 (*p *= 0.055)
	*D*_25_ (Gy)	20.08 ± 2.27	19.63 ± 2.44 (*p *= 0.008)	19.31 ± 2.32 (*p *= 0.032)
	*D*_mean_ (Gy)	13.83 ± 1.14	—	12.95 ± 1.21 (*p *= 0.013)
Heart	*D*_10_ (Gy)	16.17 ± 6.09	13.85 ± 4.75 (*p *= 0.006)	13.65 ± 5.23 (*p *= 0.003)
	*D*_5_ (Gy)	25.98 ± 7.86	20.74 ± 6.04 *(p *< 0.001)	21.11 ± 6.67 (*p *< 0.001)
	*D*_mean_ (Gy)	6.69 ± 1.39	—	6.03 ± 0.89 (*p *< 0.001)
PTV	*D*_95_ (Gy)	50.62 ± 0.81	—	50.45 ± 0.76 (*p *= 0.443)
	*D*_98_ (Gy)	49.96 ± 0.52	—	49.79 ± 0.44 (*p *= 0.315)
	*D*_2_ (Gy)	54.31 ± 1.33	—	54.11 ± 1.33 (*p *= 0.586)
	CI	0.72 ± 0.03		0.73 ± 0.02 (*p *= 0.387)
	HI	0.104 ± 0.011	—	0.095 ± 0.010 (*p *= 0.068)

**TABLE 5:** Dose–volume metrics for right lung and PTV in the manual, predicted, and re‐planning plans of right PMRMRT testing dataset.

Right PMRMRT	Dose metric	Manual	Predicted	Re‐planning
Right lung	*D*_50_ (Gy)	5.46 ± 0.51	5.26 ± 0.11 (*p *= 0.015)	4.93 ± 0.33 (*p *= 0.004)
	*D*_35_ (Gy)	10.93 ± 1.24	10.48 ± 0.40 (*p *= 0.002)	10.13 ± 0.87 (*p *= 0.009)
	*D*_25_ (Gy)	20.54 ± 2.31	19.73 ± 1.31 (*p *= 0.018)	19.22 ± 1.95 (*p *= 0.012)
	*D*_mean_ (Gy)	14.12 ± 0.64	—	13.55 ± 0.72 (*p *= 0.033)
PTV	*D*_95_ (Gy)	50.54 ± 0.66	—	50.55 ± 0.61 (*p *= 0.547)
	*D*_98_ (Gy)	50.03 ± 0.47	—	49.97 ± 0.48 (*p *= 0.415)
	*D*_2_ (Gy)	55.35 ± 1.12	—	54.79 ± 1.27 (*p *= 0.256)
	CI	0.73 ± 0.03		0.75 ± 0.02 (*p *= 0.069)
	HI	0.113 ± 0.013	—	0.0104 ± 0.012 (*p *= 0.047)

**TABLE 6:** Dose–volume metrics for left lung, heart, and PTV in the manual, predicted, and re‐planning plans of left BCRT testing dataset.

Left BCRT	Dose metric	Manual	Predicted	Re‐planning
Left lung	*D*_50_ (Gy)	3.83 ± 1.03	3.22 ± 0.31 (*p *= 0.013)	3.02 ± 0.76 (*p *= 0.055)
	*D*_35_ (Gy)	7.12 ± 2.14	7.08 ± 1.82 (*p *= 0.162)	6.81 ± 1.93 (*p *= 0.085)
	*D*_25_ (Gy)	13.30 ± 4.81	12.77 ± 3.87 (*p *= 0.048)	12.33 ± 4.32 (*p *= 0.183)
	*D*_mean_ (Gy)	10.17 ± 2.21	—	9.83 ± 1.93 (*p *= 0.086)
Heart	*D*_10_ (Gy)	8.28 ± 3.07	7.88 ± 3.62 (*p *= 0.120)	7.54 ± 3.23 (*p *= 0.074)
	*D*_5_ (Gy)	14.32 ± 7.77	13.60 ± 5.44 *(p = *0.231)	13.61 ± 6.87 (*p = *0.253)
	*D*_mean_ (Gy)	4.44 ± 1.12	—	4.31 ± 0.92 (*p = *0.187)
PTV	*D*_95_ (Gy)	43.66 ± 1.63	—	44.12 ± 1.76 (*p *= 0.843)
	*D*_98_ (Gy)	42.52 ± 1.57	—	42.89 ± 1.48 (*p *= 0.725)
	*D*_2_ (Gy)	55.49 ± 1.40	—	55.05 ± 1.37 (*p *= 0.236)
	CI	0.76 ± 0.04		0.77 ± 0.03 (*p *= 0.169)
	HI	0.298 ± 0.023	—	0.0305 ± 0.025 (*p *= 0.247)

**TABLE 7:** Dose–volume metrics for right lung and PTV in the manual, predicted, and re‐planning plans of right BCRT testing dataset.

Right BCRT	Dose metric	Manual	Predicted	Re‐planning
Right lung	*D*_50_ (Gy)	3.60 ± 1.22	3.56 ± 0.32 (*p *= 0.355)	3.21 ± 0.68 (*p *= 0.056)
	*D*_35_ (Gy)	6.61 ± 2.44	6.51 ± 0.97 (*p *= 0.282)	6.63 ± 1.65 (*p *= 0.435)
	*D*_25_ (Gy)	11.95 ± 4.83	11.94 ± 2.71 (*p *= 0.478)	11.72 ± 3.96 (*p *= 0.434)
	*D*_mean_ (Gy)	10.06 ± 2.53	—	9.82. ± 2.33 (*p *= 0.324)
PTV	*D*_95_ (Gy)	44.26 ± 1.75	—	44.32 ± 1.69 (*p *= 0.738)
	*D*_98_ (Gy)	42.85 ± 1.49	—	43.13 ± 1.55 (*p *= 0.657)
	*D*_2_ (Gy)	55.74 ± 1.25	—	55.15 ± 1.36 (*p *= 0.836)
	CI	0.78 ± 0.04		0.78 ± 0.03 (*p *= 0.869)
	HI	0.279 ± 0.021	—	0.0293 ± 0.024 (*p *= 0.447)

**FIGURE 6:**
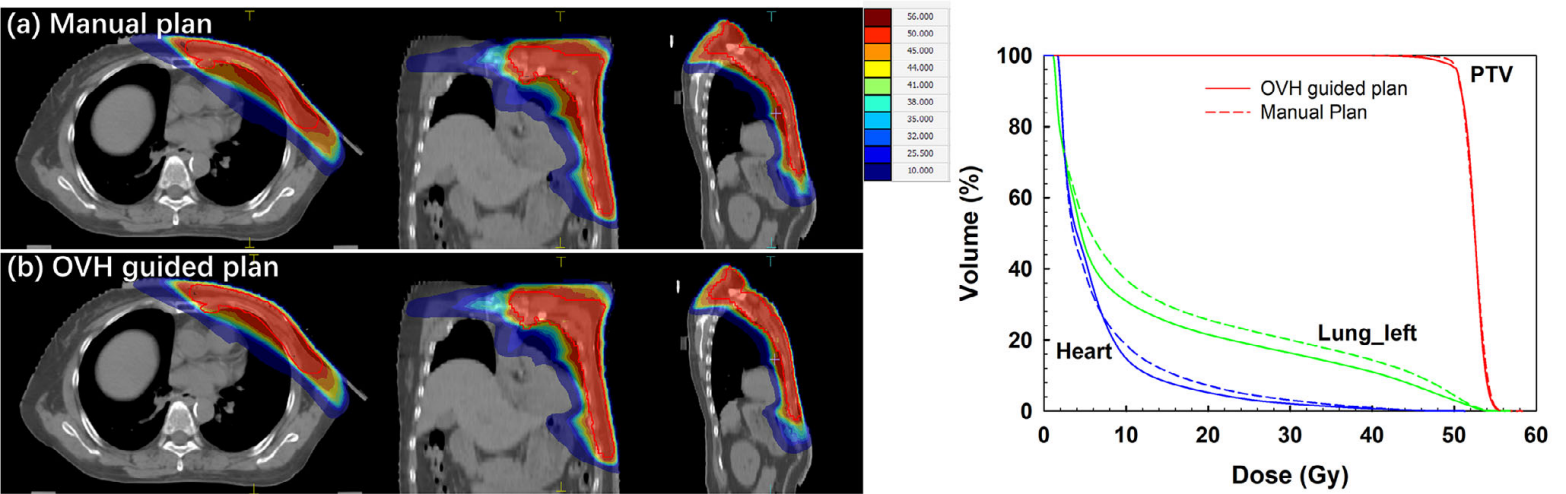
Comparison of dose distributions and corresponding DVH curves between the clinical manual plan and the automated re‐plan for a representative left‐sided PMRMRT patient.

**FIGURE 7:**
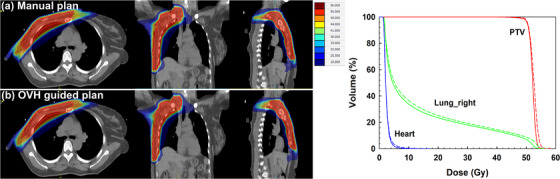
Comparison of dose distributions and corresponding DVH curves between the clinical manual plan and the automated re‐plan for a representative right‐sided PMRMRT patient.

Figures 6 and 7 illustrate the dose distributions and corresponding DVH curves for representative left‐ and right‐sided PMRMRT patients, comparing manual plans with automated re‐plans. As shown in Figure 6, the doses to both the heart and the ipsilateral lung were reduced to varying degrees in the automated plan, while the PTV coverage remained unchanged, consistent with the statistical results presented in Table 4. These findings demonstrate that the OVH‐guided approach effectively enhances the sparing of cardiopulmonary structures in PMRMRT patients. For the right‐sided PMRMRT case shown in Figure 7, the dose reduction to the ipsilateral lung was less pronounced, which may be attributable to specific anatomical variations in this particular patient. Although the re‐plan for this case exhibited a slight decrease in the PTV maximum dose, no statistically significant differences in PTV metrics were observed across the entire test dataset when compared to the original clinical plans.

## 4 DISCUSSION

The design of radiotherapy plans for breast cancer is inherently complex. While consensus guidelines provide a robust framework for target volume delineation,^13^ practical implementation is often challenged by significant inter‐patient anatomical variability. The target geometry varies substantially depending on the inclusion of the supraclavicular region and internal mammary nodes, the extension across the body midline, or the integration of a boost volume. These variations necessitate a sequence of critical planning decisions, including the selection of treatment platforms, beam energies, delivery modalities (e.g., fixed‐field IMRT vs. VMAT), and isocenter strategies.^14^ Successfully navigating these technical trade‐offs demands high‐level clinical expertise and judgment from dosimetrists.

In this study, we implemented an in‐house developed OVH method for automated treatment planning, moving beyond reliance on commercial solutions. We stratified 322 patients into four distinct models based on treatment modality and laterality (Tables 2 and 3): left‐sided PMRMRT (Model 1), right‐sided PMRMRT (Model 2), left‐sided BCRT (Model 3), and right‐sided BCRT (Model 4). For critical OARs, we observed a robust linear correlation between the OVH‐based spatial metrics (*L_x_*) and the dosimetric parameters (*D_x_*). This moderate correlation validates the feasibility of utilizing *L_x_* as a reliable predictor for achievable *D_x_* outcomes. Notably, the BCRT models exhibited slightly improved linear correlations compared to the PMRMRT models. This disparity likely stems from the relatively smaller target volumes and the higher consistency of tangential beam arrangements inherent in BCRT. In contrast, PMRMRT planning involves supraclavicular irradiation with complex beam orientations; variations in anatomical demarcation and practitioner‐specific beam setups likely introduce greater dosimetric variability in the ipsilateral lung.

By integrating these predictive models into an automated workflow, we demonstrated the capacity to generate treatment plans that significantly enhance OARs sparing. For the PMRMRT cohort, OVH‐guided re‐optimization yielded a substantial reduction in radiation exposure to the ipsilateral lung and heart compared to baseline manual plans. Crucially, this was achieved while maintaining stable PTV metrics (*D*_2_, *D*_95_, *D*_98_, CI, and HI), with only minor deviations observed in the HI for right‐sided PMRMRT. Conversely, the dosimetric outcomes for BCRT models showed no significant divergence from manual plans. This result suggests that standard clinical constraints for BCRT are already readily achievable through manual planning, potentially reaching an optimization “plateau” where further automated refinement yields diminishing returns. The PMRMRT planning is more operator‐dependent. Consequently, it is imperative to differentiate optimization constraints between BCRT and PMRMRT.^15^ This stratified, OVH‐guided approach is essential for further mitigating cardiopulmonary toxicity and improving long‐term clinical outcomes for breast cancer patients.

Our findings are in strong agreement with the growing body of literature demonstrating that knowledge‐based planning (KBP) can significantly reduce inter‐planner variability and improve dosimetric quality in radiotherapy. The limitations of traditional manual planning—being time‐consuming, iterative, and highly dependent on planner experience—are well‐documented.^3^, ^4^ Consequently, various KBP methodologies have been developed and successfully applied to breast cancer, often showing improved OAR sparing and target dose conformity.^16^, ^17^, ^18^, ^19^, ^20^ Many of these established systems, however, rely on complex statistical models of historical DVHs or machine learning algorithms. While powerful, these approaches often require a large, curated library of high‐quality plans and can function as “black boxes,” making the underlying predictive model less interpretable for clinicians and physicists. In contrast, our study contributes to the field by proposing a framework underpinned by a priori patient geometry rather than historical dosimetry. This provides a more transparent and fundamentally distinct strategy for achieving comparable clinical objectives.^21^

A key advantage of our OVH‐based framework lies in its simplicity, interpretability, and computational efficiency when compared to other automated planning models. The concept of using geometric indices to predict dose is not novel, and other descriptors have been explored.^22^, ^23^ However, our study systematically develops and validates a complete automated workflow centered on the OVH, demonstrating its practical efficacy from model building to final plan generation. The direct linear relationship we established between the OVH and DVH is intuitive and allows for the rapid prediction of achievable dose constraints immediately following organ delineation, streamlining the planning process. The significant dose reductions we achieved, particularly for the heart in left‐sided cases, directly address major clinical concerns, which established a clear link between mean heart dose and long‐term cardiac risk.^24^ By grounding our automated planning in a clear geometric principle, we provide a robust and understandable tool that translates directly to improved patient safety.

Several limitations of this study should be acknowledged. First, as a retrospective study conducted at a single institution, our models may be implicitly trained on our center's specific contouring and planning philosophies. The generalizability of these specific regression equations to other institutions with different clinical practices requires external validation. A multicenter study would be necessary to develop more universally applicable models. Second, while linear regression proved effective, the intricate relationship between anatomy and dosimetry may contain nonlinearities not captured by our models. Exploring more advanced machine learning algorithms, such as gradient boosting or neural networks, could potentially yield even higher predictive accuracy. Finally, this analysis focused primarily on the heart and ipsilateral lung as the critical OARs. Future investigations should expand these models to incorporate other relevant anatomical structures to provide a more comprehensive automated solution.

## 5 CONCLUSION

In conclusion, we have successfully developed and validated an automated treatment planning framework that translates patient‐specific anatomy into optimal dosimetric objectives via OVH predictive modeling. Our results confirmed that this automated strategy significantly reduced radiation exposure to critical thoracic structures—particularly the heart and ipsilateral lung—while maintaining dosimetric parity in target coverage compared to expert manual plans. This methodology can effectively mitigate planner‐dependent variability, and has the potential to reduce long‐term treatment‐related toxicities.

## AUTHOR CONTRIBUTIONS

Hao Lei, Xudong Xue and Dan Li selected the enrolled patients, performed the code and data analysis. Wei gave useful discussions and editing suggestions. Hongmei Zheng, Xinhong Wu, and Xudong Xue designed the study and wrote the manuscript. All authors read and approved the final manuscript.

## CONFLICT OF INTEREST STATEMENT

The authors have no conflicts to disclose.

## ETHICS STATEMENT

This study was carried out in accordance with the Declaration of Helsinki and approved by the Ethics Committee of the Hubei Cancer Hospital (No. LLHBCH2025YN‐103).

## Supporting information



Supporting Information
