# Supplementary material for: OVH‐guided planning for superior heart and lung sparing in breast cancer radiotherapy
Source: J Appl Clin Med Phys. 2026 Mar 8;27(3):e70513. doi: 10.1002/acm2.70513 (PMC12967487; doi:10.1002/acm2.70513)
Supplement: Supplementary file 1 — Supporting Information [file ACM2-27-e70513-s001.docx]

**OVH-Guided Planning for Superior Heart and Lung Sparing in Breast Cancer Radiotherapy**


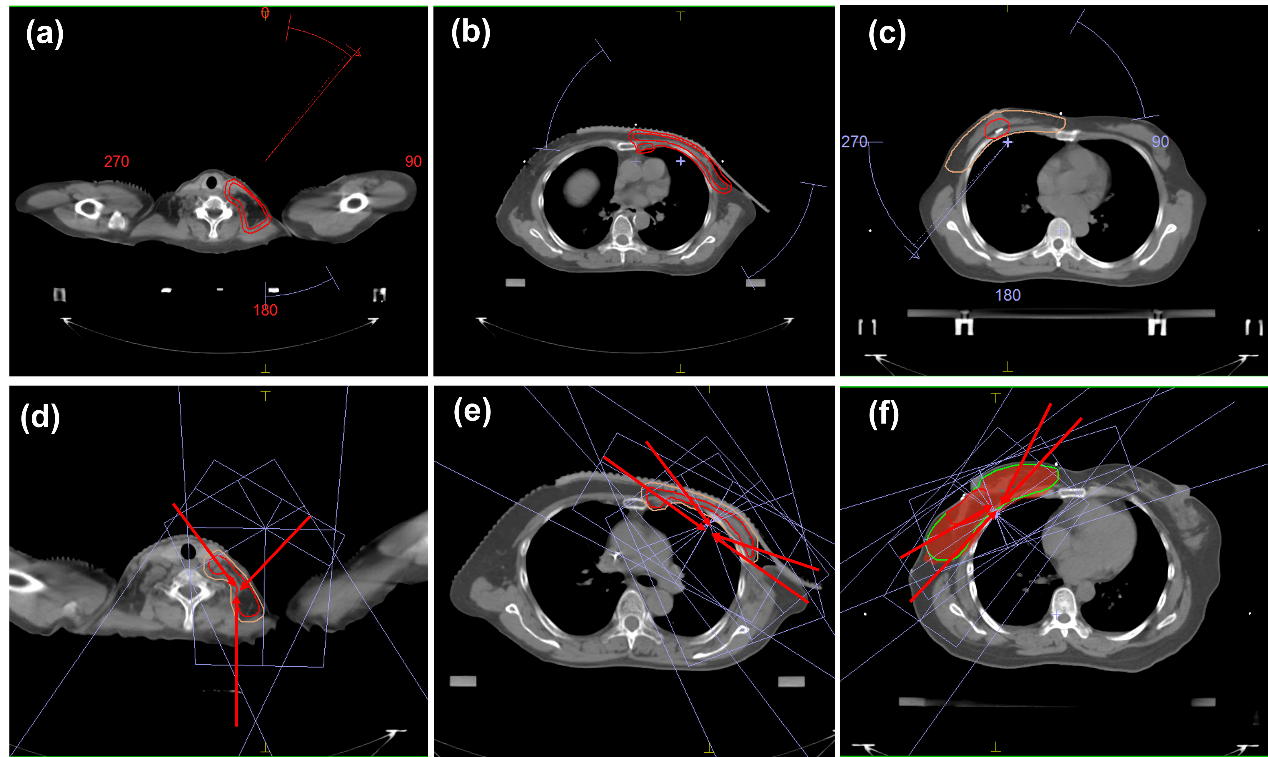


Figure S1 The details of the VMAT or IMRT techniques used in this study.

For BCRT, due to the involvement of the chest wall alone, we employ pure-tangential fields (Figure 2(c) VMAT and 2(f) IMRT techniques). In the case of PMRMRT patients, the target volumes are typically divided into two sub-regions: the supraclavicular region and the chest wall. The chest wall is irradiated using tangential fields(Figure 2(b) VMAT and 2(e) IMRT techniques), while the supraclavicular region is treated with either fixed beams (Figure 2(d)) or small partial arcs (Figure 2(a)).


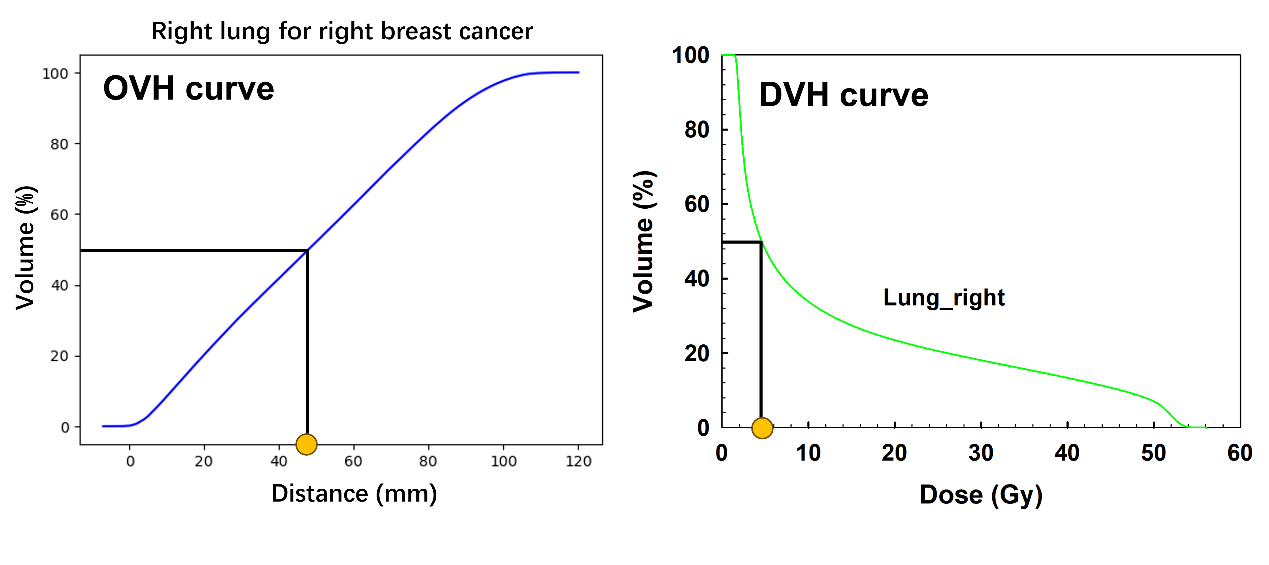


Figure S2 The definition of L_50_ at OVH curve and D_50_ at DVH curve (yellow dot).

In this study, the correlations between OVH metrics (Lx and Dx) were established using linear regression analysis across corresponding points. For instance, considering the clinical constraint for the ipsilateral lung (V_5Gy_≤50%), the metric L_50_ was defined as the isotropic expansion distance of the PTV required to encompass 50% of the lung volume. Correspondingly, D_50_ represents the dose received by 50% of the lung volume (D_50%_), serving as an indicator of lung sparing.


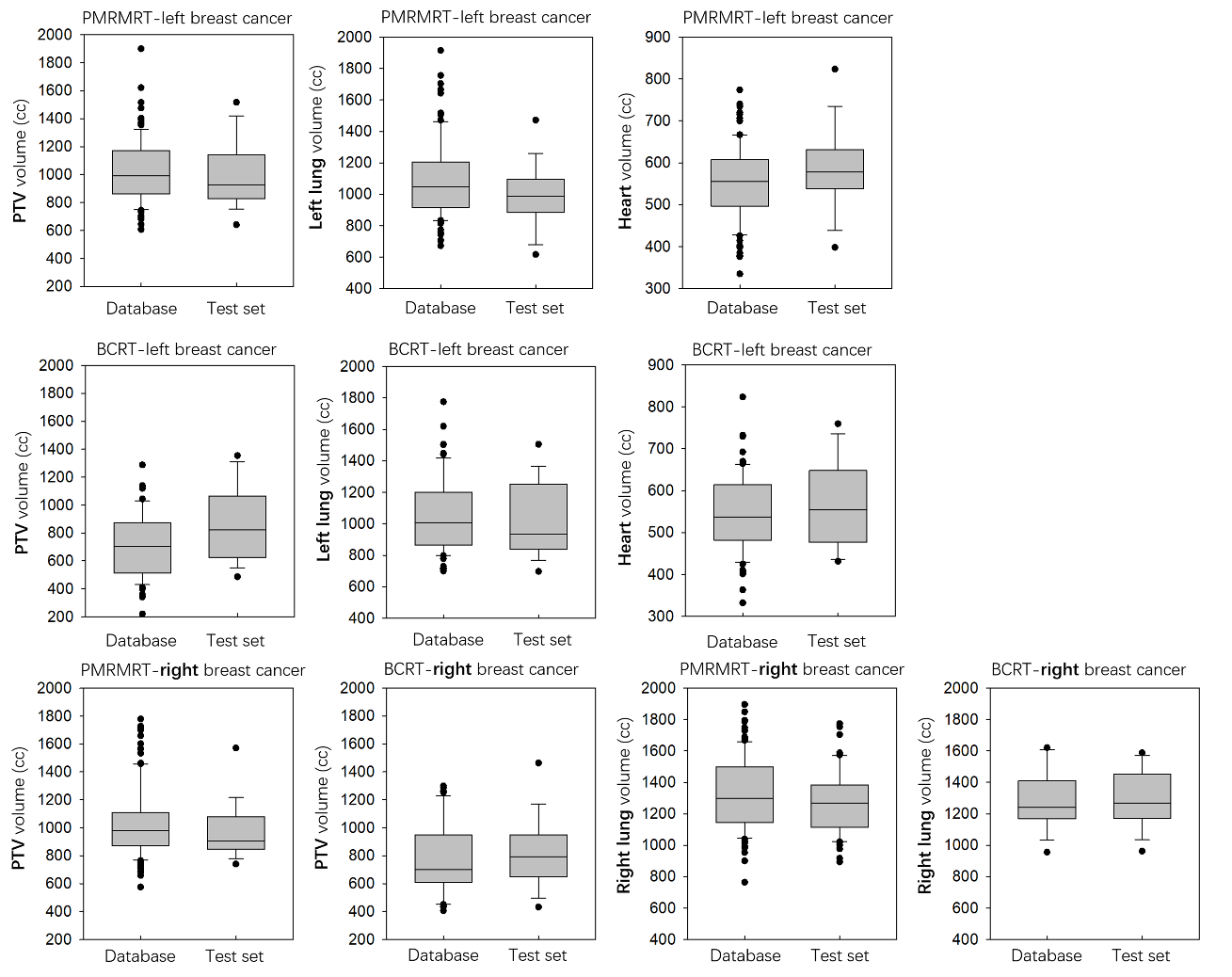


Figure S3 The boxplot of the PTV and OARs volumes from PMRMRT and BCRT cohort.
